# Reducing Misses and Near Misses Related to Multitasking on the Electronic Health Record: Observational Study and Qualitative Analysis

**DOI:** 10.2196/humanfactors.9371

**Published:** 2018-02-06

**Authors:** Neda Ratanawongsa, George Y Matta, Fuad B Bohsali, Margaret S Chisolm

**Affiliations:** ^1^ Division of General Internal Medicine Department of Medicine University of California, San Francisco San Francisco, CA United States; ^2^ University of California, San Francisco Center for Vulnerable Populations at Zuckerberg San Francisco General Hospital San Francisco, CA United States; ^3^ Division of Hospital Medicine Department of Medicine Johns Hopkins University School of Medicine Baltimore, MD United States; ^4^ Department of Psychiatry and Behavioral Sciences Johns Hopkins University School of Medicine Baltimore, MD United States

**Keywords:** electronic health records, physician-patient relations, patient safety

## Abstract

**Background:**

Clinicians’ use of electronic health record (EHR) systems while multitasking may increase the risk of making errors, but silent EHR system use may lower patient satisfaction. Delaying EHR system use until after patient visits may increase clinicians’ EHR workload, stress, and burnout.

**Objective:**

We aimed to describe the perspectives of clinicians, educators, administrators, and researchers about misses and near misses that they felt were related to clinician multitasking while using EHR systems.

**Methods:**

This observational study was a thematic analysis of perspectives elicited from 63 continuing medical education (CME) participants during 2 workshops and 1 interactive lecture about challenges and strategies for relationship-centered communication during clinician EHR system use. The workshop elicited reflection about memorable times when multitasking EHR use was associated with “misses” (errors that were not caught at the time) or “near misses” (mistakes that were caught before leading to errors). We conducted qualitative analysis using an editing analysis style to identify codes and then select representative themes and quotes.

**Results:**

All workshop participants shared stories of misses or near misses in EHR system ordering and documentation or patient-clinician communication, wondering about “misses we don’t even know about.” Risk factors included the computer’s position, EHR system usability, note content and style, information overload, problematic workflows, systems issues, and provider and patient communication behaviors and expectations. Strategies to reduce multitasking EHR system misses included clinician transparency when needing silent EHR system use (eg, for prescribing), narrating EHR system use, patient activation during EHR system use, adapting visit organization and workflow, improving EHR system design, and improving team support and systems.

**Conclusions:**

CME participants shared numerous stories of errors and near misses in EHR tasks and communication that they felt related to EHR multitasking. However, they brainstormed diverse strategies for using EHR systems safely while preserving patient relationships.

## Introduction

Clinicians spend one-third of outpatient visits using electronic health record (EHR) systems, either in silence or by multitasking [1-3]. Multitasking occurs when someone performs 2 or more tasks simultaneously. Common examples of clinician EHR multitasking are eliciting a history while entering data (voluntary multitasking) and listening to a patient’s question that arises while the clinician orders a prescription (externally prompted multitasking) [2,4]. Multitasking may increase the risk of making errors, either in communication with patients or in completing EHR tasks, such as documentation or computerized order entry [4-7]. Meanwhile, using EHR systems in silence has been associated with lower patient satisfaction [1,2]. However, delaying EHR system use until after visits may increase clinicians’ EHR workload, stress, and burnout [8,9]. This study describes the perspectives of clinicians, educators, administrators, and researchers about their experiences with misses and near misses that they felt were due to clinician multitasking while using EHR systems.

## Methods

This observational study was a thematic analysis of perspectives elicited during 3 continuing medical education (CME) courses in 2017. Participants included clinicians, clinician-educators and -administrators, and researchers attending 90-minute workshops at international health communications conferences (23 participants in Rhode Island and Maryland, USA), and clinicians and allied health professionals attending a 45-minute lecture during a course on caring for vulnerable populations (40 participants in California, USA). Workshops began with storytelling exercises about memorable times when multitasking EHR use was associated with “misses” (errors that were not caught at the time) or “near misses” (mistakes that were caught before leading to errors). Workshops and the lecture included a literature review about multitasking [1,3], video reenactments from a recent study [3], and a visioning exercise about reducing multitasking errors.

One workshop facilitator (NR) transcribed notes and quotes from participants during the interactive portions of the sessions. Two researchers (NR, MSC, or GYM) used an editing analysis style to identify “meaningful units or segments of text that both stand on their own and relate to the purpose of the study” [10]. In these data, individual quotes could represent more than one concept and be categorized by researchers under multiple different codes. We came to consensus in codes and themes and then selected representative quotes. Between the second and third session, no unique codes or themes arose, and we deemed we had reached theoretical saturation [10].

A University of California, San Francisco (UCSF) Committee on Human Research granted an exemption for this evaluation.

## Results

All workshop participants shared stories of misses or near misses (Table 1) in EHR system ordering and documentation or patient-clinician communication, wondering about “misses we don’t even know about.” Table 1 shows risk factors emerging from these stories.

Participants wanted strategies for using EHR systems during visits, while ensuring patients feel respected and heard. One participant lamented that “I’m torn between real and ideal. We would spend all day finishing notes, but [pretending to type while speaking] ‘Three sexual partners?’” Another shared that “If it has emotional value, they won’t tell me while I’m typing.”

Strategies to reduce multitasking EHR misses included (Table 2) clinician transparency when needing silent EHR use (eg, for prescribing), narrating EHR system use, patient activation during EHR system use, adapting visit organization and workflow, improving EHR system design, and improving team support and systems.

When asked for take-home intentions, 1 clinician wished to be authentic in voicing his desire to “be on the same side” with patients, acknowledging the need to use the EHR system but saying “I don’t want it to get in the way. I want you to always be able to call me back to the present.”

**Table 1:** Themes elicited from continuing medical education conference participants about misses and near misses due to multitasking on electronic health records (EHRs).

Themes and codes		Examples or quotes
**Types of misses and near misses**		
	EHR errors in ordering and documentation	Prescription electronically sent to the wrong pharmacy: “Especially when I’m calculating pediatric doses. I do it right then and don’t want to make a mistake.” Copied and pasted charting in the wrong chart: “Before you couldn’t easily get into someone else’s chart accidentally...because you would have to pull the chart and open it.”
	Communication errors	“My agenda has changed unconsciously from my agenda or my patient’s agenda before to an agenda hidden to me that affects my focus, causes me to miss things in general.” Unseen misses: “Record gives us a false sense of security that we’re capturing so much.” “I suspect I am missing things, but I hope I’m catching the ‘red flags.’”
**Risk factors**		
	Computer position	“I’m worried I don’t even know that I’m missing something because my back is to the patient.”
	EHR usability	“The buttons are so close together that I can easily click into the wrong place.” Time lags or glitches in the EHR program “I now have to find the correct lab in computer and link to a diagnosis...nothing can go forward...” “If you’re not proficient in using the computer, it’s just hard and takes more time.”
	Note content or style	“[EHR] was set up to bill, but not really designed for communicating what’s important for patient care.” “Before I could just draw a line down the pediatric physical examination boxes. Now I have to check each of multiple boxes.” Information that is not useful: “dates when medications are filled” or “inaccurate problem lists” Agenda driven by EHR: “Conversation is being driven by something else.”
	Information overload	“Prerounding helps, but there’s just so much information now.” “I find I’m going down more ‘rabbit holes’ for more information.” More graphs and tools to use
	Workflow	Keeping multiple patient charts open simultaneously Interruptions by other team members Inability to delegate: “I used to be able to ask someone to help me, but I have to do it myself now.”
	Systems issues	High volume and short visits: “I can’t imagine what the surgical specialties must do.” Perceived urgency for documentation: “Pressure for immediacy...it’s an unacceptable delay.” Concern about adding to EHR use after hours: “It will be 3 hours of my life later.”
	Provider and patient communication behaviors and expectations	“Monologue style of communication” without “open-ended invitations” Verbal “uh-huh...trumped by nonverbal body language” suggesting provider not listening Patients interrupting silent EHR use: “They think they can talk and that you can hear and listen to them, but you can’t.” Culture of screens: “It’s normal to have your face in a screen...maybe more typical more so than normal.”

**Table 2:** Strategies elicited from continuing medical education conference participants for reducing misses and near misses due to multitasking on electronic health records (EHRs).

Strategies	Examples or quotes
Awareness and transparency when silent EHR use needed	“Previewing is always helpful. There are times today when we’re going to be talking 1:1, and there are times when I’ll be using this computer. Sometimes I may even have to use the computer quietly, and while I’m doing that, you can be doing this.” “Like in the hospital, where some nurses wear a ‘stop’ sign vest for med pass—they worried about patients minding it, but when they explain it as a ‘safety measure’ then patients understand.”
Narrating during EHR use	“I talk out loud when I’m looking up test results, and I interpret the results for them. I think it helps to know what I am doing and educates them, too.”
Patient activation during EHR use	Invite History of Present Illness/Review of Systems completion together: “check these boxes with me.” Give patient education handout to review “While I’m putting this in the computer, why don’t you write down what we talked about [or] what you’re going to work on before the next visit.” “How will you remember this? Why don’t you think about that and we’ll talk afterwards.” Invite patient to “Call me back to the present.”
Visit organization and workflow	Preround before visit Avoid using the computer at the beginning of the visit or during sensitive conversations “I’m going to try to bunch things together to avoid going in and out and back in to the same section again. Like trying to do all the meds at the same time.” Ask patients to prepare for examination (eg, removing footwear for diabetes foot examination or undressing child for pediatric well visit)
Improving EHR design	Make displays of patient photos accessible for safety to reduce wrong chart documentation Reduce structured data to allow narrative documentation
Team support and systems	Voice recognition documentation support Medical scribe support: “When I saw my doctor the last time, she had a resident typing for her, and it was like a different world. She was actually looking at me.” Team support in visit documentation: “If I had help, I’d much rather have med rec before and help linking labs to ridiculous diagnoses...” “We’re being measured on patient satisfaction and quality outcomes. Both are being measured, and so both of those may be more important than doing administrative work.”

## Discussion

CME participants shared numerous stories of errors and near misses in EHR system tasks and communication that they felt related to EHR multitasking. However, they also brainstormed diverse strategies for using EHR systems safely while preserving patient relationships.

Clinicians need practical intrapersonal, interpersonal, and systems strategies to use EHR systems in mindful, relational ways. Avoiding all EHR use during patient encounters may be impossible and unsustainable, with clinicians using EHR systems over half of their workday and increasingly after clinic hours [8,9]. Meanwhile, research suggests that the risk of EHR multitasking is affected by the cognitive complexity of tasks and decisions, EHR system usability, teamwork, and clinician-patient dynamics [2-7].

Clinical multitasking predated EHR systems, which can reduce the risk of making errors by reducing the cognitive load of clinicians’ work by synthesizing and organizing information in accessible, usable formats. A 2009 Israeli study found that clinicians perceived some benefits to reducing the cognitive load of completing some clinical tasks, particularly if they perceived the EHR system to be comprehensive and usable [5]. At the same time, a danger of growing comfort and automaticity with EHR use was a risk of medication or documentation error [5]. More recent research has suggested that medication errors and adverse drug events in intensive care, hospital, and ambulatory settings may be reduced with computerized provider order entry and drug-drug interaction checking [11-13], although continuing research about errors and near misses with computerized provider order entry may yield further improvements to reduce the cognitive complexity of EHR ordering [14]. This study adds to this growing literature in the context of the rapid expansion of newer-generation EHR systems in the United States under the meaningful use incentives programs.

Clinician transparency with patients about using EHR systems—including tasks such as prescribing that require focused attention to avoid errors—may result in fewer misses while preserving patient trust and satisfaction. As professional schools implement skills-based training in patient-provider communication with EHR system use [15], trainees may be able to practice empathic ways to negotiate the need for silent EHR use and ways to detect subtle queues from patients signaling that they need the clinician’s full attention.

In addition, other systematic approaches are needed to mitigate technology-induced errors—that is, medical errors arising from a technology’s design and development, implementation and customization, and resultant human-computer interactions and sociotechnical work processes [6,7]. These include slips (errors that are corrected) and mistakes (errors that go unnoticed or uncorrected) [7]. Borycki recommended proactive and reactive methods for reducing technology-induced errors: heuristic evaluation, cognitive walkthroughs, usability testing, clinical and computer-based simulations, rapid assessment processes, ethnographies, and case studies [7].

Study limitations include the small sample size, inability to capture participant characteristics, and selection bias. Although the CME lecture participants included clinicians and allied health professionals from nonacademic clinical settings, the workshops included primarily academically based clinician-educators, clinician-administrators, and clinician-researchers. Clinicians, nurses, and other members of the health care team practicing in nonacademic environments may offer different perspectives. Recall bias and attribution bias also may have affected the findings, and we cannot be sure of the accuracy of the near misses reported by the participants or whether the near misses were due to EHR multitasking. Because we did not ask participants to name their EHR systems, we cannot be sure if their experiences involved older- or newer-generation EHR systems; however, participants who did cite their EHR systems named commercial vendors who offer products certified for meaningful use in the United States. Finally, this study did not aim to describe the patients’ perspectives, and the patients in the participants’ stories may have had different perspectives about those experiences.

Future studies should explore diverse patient perspectives about clinicians’ EHR multitasking and their strategies for bringing clinicians “back to the present.” In addition, studies should examine how these strategies affect patient-important outcomes in quality and safety.

## Abbreviations

CME: continuing medical education
EHR: electronic health record
